# Supplementary material for: A machine learning model for early candidemia prediction in the intensive care unit: Clinical application
Source: PLoS One. 2024 Sep 9;19(9):e0309748. doi: 10.1371/journal.pone.0309748 (PMC11383240; doi:10.1371/journal.pone.0309748)
Supplement: S1 Table — Definition of abbreviations: y: Years; TPN: Total parenteral nutrition; CVC: Central venous catheter; WBC: White blood cell; PCT: Procalcitonin; CRP: C-reactive protein; ICU: Intensive care unit; IQR: Interquartile range; SD: Standard deviation; *The t-test for metric variable if data are normally distributed; **the Chi-square test (big sample size) and Fisher’s exact test (small sample size) for categorical variables; ***the Mann–Whitney U-test for metric variables if data are not normally distributed. (DOCX) [file pone.0309748.s001.docx]

| S1 Table Characteristics of selected features in the external validation group. | | | |
| --- | --- | --- | --- |
|  | Candidemia (n=38) | Bacteremia (n=39) | *P* value |
| Age (y, mean (SD)) | 68.6(16.68) | 61.4(21.36） | 0.037* |
| CVC (n, %) | 24(63.2%) | 13(33.3%) | 0.012** |
| Duration of ICU stay (days, mean (SD)) | 31.5(25.40) | 4.4(4.62) | <0.001* |
| Abdominal surgery (n, %) | 23(62.2%) | 14(37.8%) | 0.031** |
| Immunosuppressive drugs (n, %) | 12(31.6%) | 5(12.8%) | 0.047** |
| Solid cancer (n, %) | 11(28.2%) | 3(7.7%) | 0.036** |
| Chemotherapy (n, %) | 7(18.4%) | 2(5.1%) | 0.087** |
| Antibiotic therapy (n, %) | 35(92.1%) | 29(74.4%) | 0.038** |
| PCT (ng/ml, median (IQR)) | 0.53(0.22, 1.78) | 4.3(0.64,14.60) | 0.002*** |
| CRP (mg/l, mean (SD)) | 85.1(53.23) | 160.6(80.07) | 0.067* |
| WBC count (10^9^/L, median (IQR)) | 9.32(5.37, 14.04) | 12.99(7.69, 15.44) | 0.072*** |
| Neutrophil count (10^9^/L, median (IQR)) | 7.81(3.61, 12.01) | 11.73(6.91, 14.23) | 0.047*** |
| Monocyte count (10^9^/L, mean (SD)) | 0.6(0.34) | 0.7(0.54) | 0.065* |
| TPN (n, %) | 20(52.6%) | 9(24.3%) | 0.017*** |
| Lymphocyte count (10^9^/L, mean (SD)) | 0.7(0.48) | 1.2(0.85) | 0.045* |
| Platelet count (10^9^/L, mean (SD)) | 143.6(118.42) | 217.3(139.91) | 0.015* |
| Hemoglobin (g/L, mean (SD)) | 96.4(17.28) | 105.7(17.84) | 0.023* |
| Total bilirubin (μmol/L, median (IQR)) | 27.50(11.80, 36.10) | 11.90(8.21, 22.10) | 0.019*** |

Definition of abbreviations: y: years; TPN: Total parenteral nutrition; CVC: central venous catheter; WBC: white blood cell; PCT: procalcitonin; CRP: C-reactive protein; ICU: intensive care unit; IQR: interquartile range; SD: standard deviation; *The t-test for metric variable if data are normally distributed; **the Chi-square test (big sample size) and Fisher’s exact test (small sample size) for categorical variables; ***the Mann–Whitney U-test for metric variables if data are not normally distributed.
