# Supplementary material for: A machine learning model for early candidemia prediction in the intensive care unit: Clinical application
Source: PLoS One. 2024 Sep 9;19(9):e0309748. doi: 10.1371/journal.pone.0309748 (PMC11383240; doi:10.1371/journal.pone.0309748)
Supplement: S2 Table — Definition of abbreviations: y: Years; TPN: Total parenteral nutrition; CVC: Central venous catheter; WBC: White blood cell; PCT: Procalcitonin; CRP: C-reactive protein; ICU: Intensive care unit; IQR: Interquartile range; SD: Standard deviation; *The t-test for metric variable if data are normally distributed; **the Chi-square test (big sample size) and Fisher’s exact test (small sample size) for categorical variables; ***the Mann–Whitney U-test for metric variables if data are not normally distributed. (DOCX) [file pone.0309748.s002.docx]

| Table S2 Comparison of selected features between internal and external validation. | | | |
| --- | --- | --- | --- |
|  | Internal validation (n=100) | External validation (n=77) | *P* value |
| Age (y, mean (SD)) | 67.5(14.65) | 64.1(19.19) | 0.114* |
| CVC (n, %) | 41(41.00%) | 37(47.44%) | 0.447** |
| Duration of ICU stay (days, mean (SD)) | 23.0(36.53) | 17.8(22.62) | 0.295* |
| Abdominal surgery (n, %) | 53(53.00%) | 37(48.05%) | 0.514** |
| Immunosuppressive drugs (n, %) | 27(27.00%) | 17(7.79%) | 0.453** |
| Solid cancer (n, %) | 21(21.00%) | 14(18.18%) | 0.641** |
| Chemotherapy (n, %) | 20(20.00%) | 9(11.69%) | 0.139** |
| Antibiotic therapy (n, %) | 84(84.00%) | 64(83.12%) | 0.875** |
| PCT (ng/ml, median (IQR)) | 2.43(0.64, 16.22) | 1.1(0.33,13.81) | 0.087*** |
| CRP (mg/l, mean (SD)) | 100.5(59.37) | 110.9(86.45) | 0.034* |
| WBC count (10^9^/L, median (IQR)) | 10.85(6.73, 16.21) | 11.25(5.78, 14.62) | 0.869*** |
| Neutrophil count (10^9^/L, median (IQR)) | 8.0(5.28, 14.16) | 9.56(4.26, 13.22) | 0.895*** |
| Monocyte count (10^9^/L, median (IQR)) | 0.55(0.23, 0.95) | 0.51(0.31, 0.79) | 0.866*** |
| TPN (n, %) | 38(38.00%) | 29(37.18%) | 0.963** |
| Lymphocyte count (10^9^/L, mean (SD)) | 0.9(0.80) | 1.1(1.35) | 0.305* |
| Platelet count (10^9^/L, mean (SD)) | 165.1(115.92) | 180.0(133.82) | 0.488* |
| Hemoglobin (g/L, mean (SD)) | 97.5(18.07) | 101.1(18.06) | 0.237* |
| Total bilirubin (μmol/L, median (IQR)) | 14.30(8.90, 34.35) | 14.35(10.43, 31.95) | 0.543*** |

Definition of abbreviations: y: years; TPN: Total parenteral nutrition; CVC: central venous catheter; WBC: white blood cell; PCT: procalcitonin; CRP: C-reactive protein; ICU: intensive care unit; IQR: interquartile range; SD: standard deviation; *The t-test for metric variable if data are normally distributed; **the Chi-square test (big sample size) and Fisher’s exact test (small sample size) for categorical variables; ***the Mann–Whitney U-test for metric variables if data are not normally distributed.
