# Supplementary material for: A machine learning model for early candidemia prediction in the intensive care unit: Clinical application
Source: PLoS One. 2024 Sep 9;19(9):e0309748. doi: 10.1371/journal.pone.0309748 (PMC11383240; doi:10.1371/journal.pone.0309748)
Supplement: S3 Table — Definition of abbreviations: TPN: Total parenteral nutrition; CRP: C-reactive protein; WBC: White blood cell; CVC: Central venous catheter. (DOCX) [file pone.0309748.s003.docx]

Table S3 Clinical Variables and Corresponding Points System

| Variable | Value Range | | Points | score |
| --- | --- | --- | --- | --- |
| Age | 10 | | 0 |  |
|  | 20 | | 1 |  |
|  | 30 | | 2 |  |
|  | 40 | | 4 |  |
|  | 50 | | 5 |  |
|  | 60 | | 6 |  |
|  | 70 | | 7 |  |
|  | 80 | | 9 |  |
|  | 90 | | 10 |  |
|  | 100 | | 11 |  |
| CVC | No | | 0 |  |
|  | Yes | | 4 |  |
| Duration of ICU stay | 0 | | 0 |  |
|  | 20 | | 2 |  |
|  | 40 | | 4 |  |
|  | 50 | | 5 |  |
|  | 60 | | 6 |  |
|  | 70 | | 7 |  |
|  | 80 | | 8 |  |
| Abdominal surgery | No | | 0 |  |
|  | Yes | | 4 |  |
| Immunosuppressive drugs | No | | 0 |  |
|  | Yes | | 5 |  |
| Solid cancer | No | | 0 |  |
|  | Yes | | 4 |  |
| chemotherapy | No | | 0 |  |
|  | Yes | | 9 |  |
| Antibiotic therapy | No | | 0 |  |
|  | Yes | | 2 |  |
| PCT | 200 | | 0 |  |
|  | 160 | | 2 |  |
|  | 120 | | 5 |  |
|  | 80 | | 7 |  |
|  | 40 | | 10 |  |
|  | 0 | | 12 |  |
| CRP | 350 | | 0 |  |
|  | 250 | | 5 |  |
|  | 150 | | 9 |  |
|  | 50 | | 12 |  |
|  | 0 | | 14 |  |
| WBC | 80 | | 0 |  |
|  | 70 | | 9 |  |
|  | 60 | | 18 |  |
|  | 50 | | 28 |  |
|  | 40 | | 38 |  |
|  | 30 | | 45 |  |
|  | 20 | | 55 |  |
|  | 10 | | 64 |  |
|  | 0 | | 72 |  |
| Neutrophils | 0 | | 0 |  |
|  | 10 | | 9 |  |
|  | 20 | | 18 |  |
|  | 30 | | 27 |  |
|  | 40 | | 38 |  |
|  | 50 | | 45 |  |
|  | 60 | | 57 |  |
|  | 70 | | 68 |  |
|  | 80 | | 76 |  |
| Monocyte | 0 | | 0 |  |
|  | 5 | | 2 |  |
|  | 10 | | 6 |  |
|  | 15 | | 8 |  |
|  | 20 | | 10 |  |
|  | 25 | | 14 |  |
|  | 30 | | 18 |  |
| TPN | No | | 0 |  |
|  | Yes | | 7 |  |
| Lymphocyte | 5 | | 0 |  |
|  | 3 | | 2 |  |
|  | 1 | | 4 |  |
| Platelet | 550 | | 0 |  |
|  | 350 | | 3 |  |
|  | 150 | | 7 |  |
|  | 0 | | 9 |  |
| [Hemoglobin](javascript:;) | 160 | | 0 |  |
|  | 130 | | 4 |  |
|  | 100 | | 8 |  |
|  | 80 | | 10 |  |
|  | 60 | | 12 |  |
|  | 40 | | 15 |  |
| Total [bilirubin](javascript:;) | 0 | | 0 |  |
|  | 100 | | 7 |  |
|  | 200 | | 12 |  |
|  | 300 | | 22 |  |
|  | 400 | | 31 |  |
|  | 500 | | 34 |  |
| Total points | | | |  |
| Total Points and Associated Risk | | | | |
| Total points | | Risk Probability | | |
| 169 | | 0.1 | | |
| 174 | | 0.3 | | |
| 177 | | 0.5 | | |
| 180 | | 0.7 | | |
| 184 | | 0.9 | | |
