# Supplementary material for: A machine learning model for early candidemia prediction in the intensive care unit: Clinical application
Source: PLoS One. 2024 Sep 9;19(9):e0309748. doi: 10.1371/journal.pone.0309748 (PMC11383240; doi:10.1371/journal.pone.0309748)
Supplement: Attachment — Submitted filename: PONE-D-24-20146_reviewer.pdf [file pone.0309748.s004.pdf]

PLOS ONE

A machine learning model for early candidemia prediction in the intensive care unit: Clinical application

--Manuscript Draft--

**Manuscript Number:**	PONE-D-24-20146
**Article Type:**	Research Article
**Full Title:**	A machine learning model for early candidemia prediction in the intensive care unit: Clinical application
**Short Title:**	candidemia prediction model in the intensive care unit
**Corresponding Author:**	Qiang Meng<br>Jining No 1 People's Hospital<br>JiNing, CHINA
**Keywords:**	Machine learning; Candidemia; Bacteremia; Random Forest algorithm; Shapley additive explanation value.
**Abstract:**	Candidemia often poses a diagnostic challenge due to the lack of specific clinical features, and delayed antifungal therapy can significantly increase mortality rates, particularly in the intensive care unit (ICU). This study aims to develop a machine learning predictive model for early candidemia diagnosis in ICU patients, leveraging their clinical information and findings. We conducted this study with a cohort of 334 patients admitted to the ICU unit at Ji Ning NO.1 people's hospital in China from Jan. 2015 to Dec. 2022. To ensure the model's reliability, we validated this model with an external group consisting of 77 patients from other sources. The candidemia to bacteremia ratio is 1:1. We collected relevant clinical procedures and eighteen key examinations or tests features to support the recursive feature elimination (RFE) algorithm. These features included total bilirubin, age, platelet count, hemoglobin, CVC, lymphocyte, Duration of stay in ICU and so on. To construct the candidemia diagnosis model, we employed random forest (RF) algorithm alongside other machine learning methods and conducted internal and external validation with training and testing sets allocated in a 7:3 ratio. The RF model demonstrated the highest area under the receiver operating characteristic (AUC) with values of 0.87 and 0.83 for internal and external validation, respectively. To evaluate the importance of features in predicting candidemia, Shapley additive explanation (SHAP) values were calculated and results revealed that total bilirubin and age were the most important factors in the prediction model. This advancement in candidemia prediction holds significant promise for early intervention and improved patient outcomes in the ICU setting, where timely diagnosis is of paramount crucial.
**Order of Authors:**	Qiang Meng
	Bowang Chen
	Yingyuan Xu
	Qiang Zhang
	Ranran Ding
	Zhen Ma
	Zhi Jin
	Shuhong Gao
	Feng Qu
**Additional Information:**	
**Question**	**Response**
**Financial Disclosure**<br><br>Enter a financial disclosure statement that	Yes

Powered by Editorial Manager® and ProduXion Manager® from Aries Systems Corporation

describes the sources of funding for the work included in this submission. Review the submission guidelines for detailed requirements. View published research articles from *PLOS ONE* for specific examples.<br><br>This statement is required for submission and **will appear in the published article** if the submission is accepted. Please make sure it is accurate.<br><br>**Funded studies**<br>Enter a statement with the following details:<br>• Initials of the authors who received each award<br>• Grant numbers awarded to each author<br>• The full name of each funder<br>• URL of each funder website<br>• Did the sponsors or funders play any role in the study design, data collection and analysis, decision to publish, or preparation of the manuscript?<br><br>Did you receive funding for this work?	
Please add funding details.<br>as follow-up to "**Financial Disclosure**<br><br>Enter a financial disclosure statement that describes the sources of funding for the work included in this submission. Review the submission guidelines for detailed requirements. View published research articles from *PLOS ONE* for specific examples.<br><br>This statement is required for submission and **will appear in the published article** if the submission is accepted. Please make sure it is accurate.	Key R&D Program of Jining, grant number 2023YXNS151.

**Funded studies**<br>Enter a statement with the following details:<br>• Initials of the authors who received each award<br>• Grant numbers awarded to each author<br>• The full name of each funder<br>• URL of each funder website<br>• Did the sponsors or funders play any role in the study design, data collection and analysis, decision to publish, or preparation of the manuscript?<br><br>Did you receive funding for this work?"	
Please select the country of your main research funder (please select carefully as in some cases this is used in fee calculation).<br>as follow-up to "**Financial Disclosure**<br><br>Enter a financial disclosure statement that describes the sources of funding for the work included in this submission. Review the submission guidelines for detailed requirements. View published research articles from *PLOS ONE* for specific examples.<br><br>This statement is required for submission and **will appear in the published article** if the submission is accepted. Please make sure it is accurate.<br><br>**Funded studies**<br>Enter a statement with the following details:<br>• Initials of the authors who received each award<br>• Grant numbers awarded to each author<br>• The full name of each funder<br>• URL of each funder website<br>• Did the sponsors or funders play any role in the study design, data collection and analysis, decision to publish, or preparation of the manuscript?<br><br>Did you receive funding for this work?"	CHINA - CN
**Competing Interests**	The authors have declared that no competing interests exist.

Use the instructions below to enter a competing interest statement for this submission. On behalf of all authors, disclose any competing interests that could be perceived to bias this work—acknowledging all financial support and any other relevant financial or non-financial competing interests.<br><br>This statement is required for submission and **will appear in the published article** if the submission is accepted. Please make sure it is accurate and that any funding sources listed in your Funding Information later in the submission form are also declared in your Financial Disclosure statement.<br><br>View published research articles from *PLOS ONE* for specific examples.<br><br>**NO authors have competing interests**<br><br>Enter: *The authors have declared that no competing interests exist.*<br><br>**Authors with competing interests**<br><br>Enter competing interest details beginning with this statement:<br><br>*I have read the journal's policy and the authors of this manuscript have the following competing interests: [insert competing interests here]*<br><br>* typeset	
**Ethics Statement**<br><br>Enter an ethics statement for this submission. This statement is required if the study involved:<br><br>• Human participants<br>• Human specimens or tissue<br>• Vertebrate animals or cephalopods<br>• Vertebrate embryos or tissues	The study protocol received approval from the Human Ethics Review Committee of Ji Ning NO.1 people's Hospital, assigned the reference number KYLL-202308-134. The ethics review board at Ji Ning No.1 people's Hospital granted an exemption from the acquisition of informed consent, given the retrospective nature of the study.

• Field research<br><br>Write "N/A" if the submission does not require an ethics statement.<br><br>General guidance is provided below. Consult the submission guidelines for detailed instructions. **Make sure that all information entered here is included in the Methods section of the manuscript.**	

Powered by Editorial Manager® and ProduXion Manager® from Aries Systems Corporation

**Format for specific study types**<br><br>**Human Subject Research (involving human participants and/or tissue)**<br>• Give the name of the institutional review board or ethics committee that approved the study<br>• Include the approval number and/or a statement indicating approval of this research<br>• Indicate the form of consent obtained (written/oral) or the reason that consent was not obtained (e.g. the data were analyzed anonymously)<br><br>**Animal Research (involving vertebrate animals, embryos or tissues)**<br>• Provide the name of the Institutional Animal Care and Use Committee (IACUC) or other relevant ethics board that reviewed the study protocol, and indicate whether they approved this research or granted a formal waiver of ethical approval<br>• Include an approval number if one was obtained<br>• If the study involved *non-human primates*, add *additional details* about animal welfare and steps taken to ameliorate suffering<br>• If anesthesia, euthanasia, or any kind of animal sacrifice is part of the study, include briefly which substances and/or methods were applied<br><br>**Field Research**<br><br>Include the following details if this study involves the collection of plant, animal, or other materials from a natural setting:<br>• Field permit number<br>• Name of the institution or relevant body that granted permission	
**Data Availability**<br><br>Authors are required to make all data underlying the findings described fully available, without restriction, and from the time of publication. PLOS allows rare exceptions to address legal and ethical concerns. See the PLOS Data Policy and FAQ for detailed information.	Yes - all data are fully available without restriction

A Data Availability Statement describing where the data can be found is required at submission. Your answers to this question constitute the Data Availability Statement and **will be published in the article**, if accepted.<br><br>**Important:** Stating 'data available on request from the author' is not sufficient. If your data are only available upon request, select 'No' for the first question and explain your exceptional situation in the text box.<br><br>Do the authors confirm that all data underlying the findings described in their manuscript are fully available without restriction?	
**Describe where the data may be found in full sentences. If you are copying our sample text, replace any instances of XXX with the appropriate details.**<br><br>• If the data are **held or will be held in a public repository**, include URLs, accession numbers or DOIs. If this information will only be available after acceptance, indicate this by ticking the box below. For example: *All XXX files are available from the XXX database (accession number(s) XXX, XXX.).*<br>• If the data are all contained **within the manuscript and/or Supporting Information files**, enter the following: *All relevant data are within the manuscript and its Supporting Information files.*<br>• If neither of these applies but you are able to provide **details of access elsewhere**, with or without limitations, please do so. For example:<br><br>*Data cannot be shared publicly because of [XXX]. Data are available from the XXX Institutional Data Access / Ethics Committee (contact via XXX) for researchers who meet the criteria for access to confidential data.*<br><br>*The data underlying the results presented in the study are available from (include the name of the third party*	All relevant data are within the manuscript and its Supporting Information files.

and contact information or URL).<br>• This text is appropriate if the data are owned by a third party and authors do not have permission to share the data.<br><br>* typeset	
Additional data availability information:	

Manuscript Click here to access/download;Manuscript;5.17.docx

Article

A machine learning model for early candidemia prediction in the intensive care unit: Clinical application

Qiang Meng [1], Bowang Chen[1], Yingyuan Xu[2], Qiang Zhang[2], Ranran Ding[1], Zhen Ma[1], Zhi Jin[1], Shuhong Gao[1*], and Feng Qu [1*]

1 Department of Intensive Care Unit, Affiliated Jining No.1 People's Hospital of Shandong First Medical University, Jiankang Road No.6, Jining City, Shandong Province, People's Republic of China.

2 Pulmonary and Critical Care Medicine, Tengzhou Central People's Hospital, Tengzhou City, Shandong Province, People's Republic of China.

*Corresponding author

E-mail: rmyyzzeq@163.com (F. Q.);

E-mail: mengqiang1985@126.com(S. G.).

Abstract

Candidemia often poses a diagnostic challenge due to the lack of specific clinical features, and delayed antifungal therapy can significantly increase mortality rates, particularly in the intensive care unit (ICU). This study aims to develop a machine learning predictive model for early candidemia diagnosis in ICU patients, leveraging their clinical information and findings. We conducted this study with a cohort of 334 patients admitted to the ICU unit at Ji Ning NO.1 people's hospital in China from Jan. 2015 to Dec. 2022. To ensure the model's reliability, we validated this model with an external group consisting of 77 patients from other sources. The candidemia to bacteremia ratio is 1:1. We collected relevant clinical procedures and eighteen key examinations or tests features to support the recursive feature elimination (RFE) algorithm. These features included total bilirubin, age, platelet count, hemoglobin, CVC, lymphocyte, Duration of stay in ICU and so on. To construct the candidemia diagnosis model, we employed random forest (RF) algorithm alongside other machine learning methods and conducted internal and external validation with training and testing sets allocated in a 7:3 ratio. The RF model demonstrated the highest area under the receiver operating characteristic (AUC) with values of 0.87 and 0.83 for internal and external validation, respectively. To evaluate the importance of features in predicting candidemia, Shapley additive explanation (SHAP) values were calculated and results revealed that total bilirubin and age were the most important factors in the prediction model. This advancement in candidemia prediction holds significant promise for early intervention and improved patient outcomes in the ICU setting, where timely diagnosis is of paramount crucial.

Keywords: Machine learning; Candidemia; Bacteremia; Random Forest algorithm; Shapley additive explanation value.

Introduction

Fever is a common symptom among patients admitted to the intensive care unit (ICU), often attributed to infections caused by variable microorganisms. *Candida* spp. is the leading cause of invasive fungal infections, ranking as the fourth most common microorganisms responsible for bloodstream infections (BSIs) [1]. Both invasive fungal infections and candidemia predominantly affect critically ill patients, with up to one-third episodes of candidemia cases occurring within the ICU[2]. Candidemia usually causes life-threatening conditions, resulting in increased mortality rate, healthcare costs, and prolonged ICU stays [3, 4]. However, distinguishing candidemia from bacteremia is challenging due to the lack of specific clinical symptoms and presentations [5, 6]. The current gold standard for candidemia diagnosis is a positive blood culture test[7]. However, blood culture tests have low sensitivity and require extended incubation times, potentially delaying antifungal therapy[8]. Therefore, physicians usually need to decide whether to wait [illegible]od culture result which may delay initiation of antifungal therapy and prescribe empirical antifungal drugs when they suspect candidemia infection based on their experience. Retrospective studies have showed that such de-

lays can substantially enhance mortality rates, with percentages rising from 35% to 80%[9, 10]. Ad[illegible]sely, initiate empirical antifungal treatment in early time would induce antifungal resistance which has become a significant global public health threat.

New diagnostic biomarkers such as (1, 3)-β-D-glucan (BGD) and galactomannan (GM), glycan antigens derived from the fungal cell wall, or antibody recognizing mannan, have been introduced in clinical practice[6]. However, these biomarker tests are often costly and require complex infrastructure, limiting hospitals' ability to conduct such around-the-clock tests[11]. So, they may not provide early diagnosis of candidemia in ICU patients, leaving clinicians without timely information when suspecting a candidemia infection[12]. Furthermore, biomarkers could not accurately predict candidemia in many conditions. BGD performed high percentage of false-positive results due to transfusion of human blood products, hemodialysis, infection by some Gram-positive bacteria, use of certain beta-lactam antibiotics, cellulose dressings or enteral nutrition, and disruptions of gastrointestinal (GI) tract integrity[13, 14].Therefore, the demand for the development of more rapid and accurate approaches to differentiate candidemia from bacterial infections is not only evident but also urgent, such approaches could aid ICU physicians in making informed decision regarding the initiation of antifungal therapy [15, 16].

Recently, clinical decision support based on machine learning (ML) have received increasing attention on candidemia early prediction. ML techniques possess a unique capacity to process vast quantities of data, enabling them employ mathematical models in conjunction with training modules to grasp intricate patterns within clinical datasets. This capability equips ML to excel in tasks such as pattern detection and differentiation[17]. Previous studies have unveiled the potential of ML in early candidemia diagnosis. Bhavani SV *et al.*[16] trained a gradient boosting machine (GBM) model for early detection of candidemia in high and low risk groups. Their model performed the traditional logistic regression model, demonstrating the effectiveness of ML approaches with a higher area under the receiver operating characteristic (AUC). Similarly, Yoo J *et al.* [18] develops the CanDETEC model, utilizing random forest (RF) algorithms for candidemia risk prediction in patients with malignancy, and validates its efficacy through a single-center retrospective study. applied random forest (RF) models for the early prediction of candidemia in cancer patients. Atamna A *et al.*[19] and Gao Y *et al.*[20]constructed a logistic regression (LR) model to pinpoint risk factors of candidemia in internal medicine wards, offering valuable insights for the clinical management of patients with candidemia and bacteremia. However, the aforementioned studies primarily focused general populations of patients outside the ICU setting. Risk factors for *Candida* bloodstream infections in adult patients have been extensively described and identified, often observed in hospitalized patients. Nevertheless, critically ill ICU patients often present with distinct risk factors that are less applicable to distinguishing from bacteremia patients. Furthermore, the majority of studies have relied on traditional linear models to construct their predictive models, which offer only moderate predictive accuracy even in the best performing models.

The demand for a more accurate and rapid approach to differentiate candidemia from bacteremia in ICU patients has grown, which will guide timely and effective therapeutic interventions in clinical settings. This study focuses on developing an early prediction model for fungal bloodstream infections in ICU patients, employing various machine

learning algorithms. Through extensive retrospective analysis and external validation, the study validates the effectiveness and generalizability of the models. Additionally, the research elucidates the contribution of individual features in the model using SHAP values and constructs a predictive nomogram for diagnosing candidemia, providing clinicians with a valuable decision-making tool.

Materials and Methods

Human Ethics

This study adhered to the guidelines set forth in the TRIPOD (Transparent Reporting of a Multivariable Prediction Model for Individual Prognosis or Diagnosis)[21] for the construction of the multivariable prediction model and its subsequent validation. The study protocol received approval from the Human Ethics Review Committee of Ji Ning NO.1 people's Hospital, assigned the reference number KYLL-202308-134. The ethics review board at Ji Ning No.1 people's Hospital granted an exemption from the acquisition of informed consent, given the retrospective nature of the study. All patients' information was handled confidentially during the data collection and manuscript preparation.

Patient inclusion and exclusion criteria

Clinical data for model development and internal validation were retrospectively collected from the electronic medical record system at Ji Ning No. 1 People's Hospital, spanning from Jan. 2015 to Dec. 2022. The clinical data for this study were accessed from September 2023 to October 2023. Patients in the candidemia group were included if they met the following criteria: (1) aged ≥ 18 years; (2) requirement for intensive care; (3) expected duration of stay in ICU >48 hours; and (4) at least one blood peripheral positive for Candida spp. Candidemia group excluded criteria: (1) age < 18 years; (2) patients who co-infected with bacteremia within 7 days before and after the detection of candidemia; (3) patients with fungal infections other than those caused by Candida species, or (iv) patients who died within 48 hours. (4) patients who were pregnant or lactating. Control group patients were selected based on the following criteria: diagnosis of bacteremia with matched age (± 5 years), sex and date of hospital admission at the time of the first positive blood culture. A 1:1 ratio of controls to candidemia cases. To ensure a comparable periods of risk exposure for both groups, each control patient had a hospitalization duration similar to the time at risk of cases, defined as the number of days from hospital admission to the occurrence of candidemia. This matching strategy aims to create a balanced and representative dataset for the analysis[22].

For external validation, clinical data were obtained from Teng Zhou Central People's Hospital, covering the period from Jan. 2020 to Jun. 2023. The external validation data for this study were accessed from January 2024 to February 2024.

Data collection

We determined the potential risk factors for Candida infection through reviewing ~~the~~ past literature and discussions among the experts in the research team. BDG and GM tests, as new biomarkers, have been rarely used in the past, so we did not conduct those as risk factors in our study. Data collected at baseline encompassed various characteristics, including the duration of stay in ICU, antibiotic usage within the previous two weeks, presence of solid cancer, diabetes, history of chemotherapy, recent surgical procedures, use of central venous catheter (CVC), receipt of total parenteral nutrition (TPN), immunosuppressant treatments and other clinical laboratory data. Prior to analysis, we applied data processing techniques. Cases with missing data excessing 50% of features were excluded from the dataset. To ensure the quality and completeness of the dataset for subsequent analysis, missing values in the remaining cases were imputed using the mean value of the training set and testing set, respectively.

Microbiological test

Blood culture was processed using the automated blood culture system (Bio-Merieux SA, Marcy l'etoile, France). Fungal isolates and bacterial isolates were cultured at 35 ℃for 48-72 hours. Simultaneously, we conducted gram staining and microscopic examination. Strain identification was carried out using the VITEK2 Compact system (Bio-Merieux SA, Marcy l'etoile, France) for both fungal and bacterial isolates.

Feature selection techniques

Feature selection is a crucial process aimed at identifying the most relevant subset of variables within a dataset. This practice serves to reduce the number of features, enhance the model's generalization ability, and mitigate the risk of overfitting [23]. While machine learning algorithms in our study can automatically rank features of importance, they may occasionally introduce a small number of redundant features due to the inherent randomness in feature selection [24]. To address this problem, we employed recursive feature elimination (RFE) for feature selection. RFE involves the iterative removing of the least important features, allowing us to identify the optimal feature combination that can improve overall generalization performance [25].

Model development and external validation

We used a range of machine learning algorithms, including extreme gradient boosting (XGB), logistic regression (LR), support vector machines (SVM), recurrent neural network (RNN) and random forest (RF), to establish candidemia prediction models with the selected variables. These models underwent ten-fold cross-validation on the training set to determine the optimal hyperparameters. For cross-validation, the entire dataset was randomly divided into ten groups. In each iteration, seven groups of patients were assigned to the training cohort, while the remaining

three groups of patients were assigned to the testing cohort. This random assignment was repeated ten times, each time using a different combination of data groups as training and testing sets, resulting in ten-fold cross-validation.

External validation was performed using an independent set of electronic records of healthy individuals obtained from Teng Zhou Central People's Hospital spanning from 2020 to 2023. Through machine learning algorithm of RFE, we have obtained features for constructing an early-stage candidemia predict model. Then, we collected data of the selected features from Teng Zhou Central People's Hospital for external validation of candidemia predict model.

Evaluating performance and interpretation of models

We use receiver operating characteristic curve (ROC) to predict power of all the models for candidemia diagnosis. The area under the ROC curve (AUC) served as a metric to assess their capacity to distinguish candidemia from bacteremia. The best predict model was defined as the one with the highest AUC value. Furthermore, we assessed performance using metrics such as accuracy, precision, recall, and F1-score to comprehensively evaluate the predictive model's effectiveness [26]. Score and accuracy were calculated using a threshold of 0.5. Model calibration was visualized through the calibration curve [27]. These curves plot the mean decile prediction probability for patients in each group. Calibration results were evaluated by examining the proximity of the calibration curve to the identity line (y = x), representing the standard calibration. In addition, we conducted a decision curve analysis to evaluate the potential benefits of clinical decision- making based on RF model predictions.

Given that machine learning model are often considered as black-boxes, understanding the impact of each risk variable on predict model can be challenging. To address this problem, we used Shapley additive explanation (SHAP) value [28] to interpret the predictive models and judge the contribution of each variable. SHAP value was conducted using the "create_explainer" and "shap" function in "shapper" R package (version 4.3.2). Finally, we used nomograms to estimate probability of individual candidemia infection using the "nomogram " function in the "rms" R package (version 4.3.2)[29]. The nomogram analysis considered multiple variables while offers a more accurate prediction and enhances decision-making for candidemia prediction.

Statistical analysis

Continues data were presented as mean± standard deviation (SD) or median (interquartile range, IQR), while categorical data were reported as frequencies and percentages. Univariate analyses were conducted, with continues data analyzed using independent t-test or non-parametric tests, and categorical data analyzed using the Chi-square or Fisher's exact test. For the assessed models, the area under the receiver operating characteristic curve (AUROC) was applied to assess performance in differentiating candidemia patients from bacteremia populations. The Statistical analyses were performed using IBM SPSS Statistics for Windows, Version 25.0. A significance level of p <0.05 was considered statistically significant.

Results

Significant differences in patient parameters

In this study, we included a total of 334 patients, comprising 167 patients with candidemia and 167 with bacteremia, to construct a risk prediction model for early candidemia diagnosis in ICU setting. The patient cohort was divided into a training set that consisted of 70% of the patients (234 patients), and a testing set that comprised the remaining 30% (100 patients). Additionally, data from 77 patients, with 38 diagnosed with candidemia and 39 with bacteremia, were recruited from Teng Zhou Central People's Hospital to serve as an external validation set. The procedure for patient’s selection is detailed in Fig 1.

Fig 1. The flow chat of candidemia and bacteremia patients which were selected in ICU for model development, internal validation and external validation. (A)Patients select from Jining NO. 1 people’s hospital for model development and internal validation. (B) Patients select from Teng Zhou People's Hospital for external validation.

Comparisons of the baseline features between the candidemia and bacteremia group were provided in Table1. As shown in Table1, age is significantly difference between candidemia and bacteremia patients. There is no statistically significant difference in comorbidities between candidemia and bacteremia patients. The key characteristics of the patients in the training and testing dataset are presented in Table 2. As shown in Table 2, several features, including age, presence of central venous catheter (CVC), platelet count, use of total parenteral nutrition (TPN), and duration of stay in the ICU, exhibited significant differences between patients with candidemia and those with bacteremia patients, both in training and test sets. However, there was no difference in gender, presence of fever, alanine transaminase (ALT), aspartate transaminase (AST), albumin, glutamyl transpeptidase (GGT), total bilirubin, creatinine, diabetes, history of solid cancer, chemotherapy, use of immunosuppressive drugs, antibiotic therapy, prior abdominal surgery, white blood count (WBC), neutrophils count, lymphocyte count, C-reactive protein (CRP), procalcitonin (PCT) levels, and hemoglobin level between candidemia and bacteremia patients. A detailed breakdown of the selected features for candidemia and bacteremia in external validation can be found in S1 Table. The comparison of selected features between internal and external validation can be found in S2 Table, WBC and CRP exhibited significant difference.

Table 1. Baseline features between the candidemia and bacteremia groups.

	Candidemia (n=167)	Bacteremia (n=167)	*P* value

Gender (male, n (%))	112(67.6%)	106(63.7%)	0.367**
Age (y, mean (SD))	70.6（17. 49）	64.5(16.85)	0.036*
CHD disease (n (%))	48(28.7%)	46(27.5%)	0.852**
High blood pressure disease (n (%))	68(40.7%)	74(44.3%)	0.623**
Diabetes (n (%))	58(34.7%)	48(28.7%)	0.239**
Chronic pulmonary disease (n (%))	48(28.7%)	46(27.5%)	0.851**
Liver disease (n (%))	8(4.79%)	12(7.14%)	0.363**
Renal disease (n (%))	15(45.5%)	18(54.5%)	0.582**
Cerebrovascular disease (n (%))	49(29.3%)	34(20.3%)	0.118**

Definition of abbreviations: y: years; CHD: coronary atherosclerotic heart disease; *The t-test for metric variable if data are normally distributed; **the Chi-square test (big sample size) and Fisher's exact test (small sample size) for categorical variables.

Table 2. Patient parameters between candidemia and bacteremia in training and testing sets.

	Infection patients(n=334)					
	Training set(n=234)		*P* value	Testing set (n=100)		*P* value
	Candidemia (n=113)	Bacteremia (n=121)		Candidemia (n=54)	Bacteremia (n=46)	
Gender (male, n (%))	74（65.4%）	78(64.5%)	0.570**	31(57.4%)	29(63.0%)	0.551**
Age (y,mean (SD))	70.2(16.72)	64.2(16.31)	0.027*	71.7(12.81)	65.8(15.56)	0.019*
Fever (℃, mean (SD))	38.2(0.68)	38.6(0.54)	0.795*	38.2(0.68)	38.6(0.56)	0.124*
ALT (U/L, median (IQR))	25.9(9.5,58.2)	23.9(15.1,31.5)	0.779***	28.5(12.58,91.78)	30.95(13.73,60.38)	0.828***
AST (U/L, median (IQR))	28.0(17.5, 69.9)	47.5(18.6, 97.0)	0.221***	43.6(27.3, 73.7)	33.2(21.1, 68.8)	0.258***
Albumin (g/l, mean (SD))	28.5(5.60)	29.4(5.11)	0.339*	28.1(6.55)	31.0(5.92)	0.068*
GGT (U/L，median (IQR))	55.0(30.1,107.6)	49.0(25.5,135.5)	0.979***	58.0(37.50,111.5)	42.95(22.0, 115.5)	0.079***

Total bilirubin(μmol/L, median (IQR))	26.8(10.7, 41.7)	15.5(9.1, 26.9)	0.037***	27.3(8.9, 45.8)	10.8(8.9, 16.1)	0.028***
Creatinine (μmol/L, median (IQR))	91.4(68.3, 72.3)	175.0(43.0,241.8)	0.046***	99.0(63.0, 138.0)	172.5(52.3, 237.0)	0.035***
Diabetes (n, %)	38(33.6%)	35(28.9%)	0.481**	20(37.0%)	13(28.3%)	0.398**
Solid cancer (n, %)	32(28.3%）	18(14.9%)	0.012**	15(27.8%)	6(13.0%)	0.071**
chemotherapy (n, %)	26(23.0%)	14(11.6%)	0.094**	14(25.9%)	6(13.0%)	0.126**
Immunosuppressive drugs (n, %)	35(31.0%)	21(16.4%)	0.008**	19(35.2%)	8(17.4%)	0.046**
Antibiotic therapy (n, %)	105(92.9%)	100(82.6%)	0.017**	49(90.7%)	35(76.1%)	0.046**
CVC (n, %)	68(60.2%)	31(25.6%)	<0.001**	31(57.4%)	10(21.7%)	0.001**
Abdominal surgery (n, %)	70(61.95%)	55(45.45%)	0.012***	34(63.0%）	19(41.3%)	0.044***
WBC count (109/L, median (IQR))	9.9(6.5,17.3)	17.4(7.9,20.8)	0.041***	11.2(7.6, 20.6)	19.1(6.5, 24.7)	0.043***
CRP (mg/l, mean (SD))	85.3(55.32)	130.4(80.61)	0.014*	91.6(51.57)	138.6(66.45)	0.034*
PCT (ng/ml, median (IQR))	1.9(0.7,10.9)	3.9(0.3,17.3)	0.034***	2.0(0.6, 14.0)	3.3(0.8, 10.4)	0.044***
Neutrophils count (109/L, median (IQR))	7.0(5.6, 14.9)	10.3(6.1, 14.2)	0.344***	7.31(4.90, 10.72)	10.27(5.75, 17.77)	0.101***
Lymphocyte count (109/L, mean (SD))	0.8(0.62)	1.3(0.73)	0.036*	0.67(0.60)	1.23(0.90)	0.048*
Platelet count (109/L, mean (SD))	139.1(109.71)	186.8(113.31)	0.011*	123.6(97.97)	208.4(119.52)	0.003*
Monocyte count (109/L, median (IQR))	0.35(0.24, 0.79)	0.66(0.36, 0.88)	0.023***	0.34(0.19, 0.73)	0.66(0.43, 1.12)	0.425***

Hemoglobin(g/L, mean (SD))	84.1(19.22)	100.9(21.52)	0.028***	79.9(16.41)	98.9(19.53)	0.027***
TPN (n, %)	62(54.9%)	27(22.3%)	<0.001**	28(51.9%)	10(21.7%)	0.004**
Duration of ICU stay (days, mean (SD))	24.0(21.6)	14.4(15.5)	0.046*	27.5(37.1)	16.7(35.9)	0.030*

Definition of abbreviations: y: years; ALT: alanine transaminase; AST: aspartate transaminase; GGT: glutamyl transpeptidase; TPN: Total parenteral nutrition; CVC: central venous catheter; WBC: white blood cell; PCT: procalcitonin; CRP: C-reactive protein; ICU: intensive care unit; IQR: interquartile range; SD: standard deviation; *The t-test for metric variable if data are normally distributed; **the Chi-square test (big sample size) and Fisher's exact test (small sample size) for categorical variables; ***the Mann–Whitney U-test for metric variables if data are not normally distributed.

Model development and selected features

To identify the most relevant features for our prediction model, the Recursive Feature Elimination (RFE) algorithm, a tree-based method, was utilized to rank the best feature combination [30]. Finally, the most significant 18 features were selected according to their importance, which include total bilirubin, age, platelet count, hemoglobin, CVC, lymphocyte, duration of stay in ICU, neutrophils, monocyte, antibiotic therapy, PCT, CRP, WBC, immunosuppressive drugs, TPN, abdominal surgery, chemotherapy and solid cancer (Fig 2).

Fig 2. Feature recursive optimization. The dotted line shows the highest accuracy is achieved with 18 features. The horizontal axis is the number of feature selections, and the vertical axis is the prediction accuracy.

Model performance for predicting candidemia

We employed multiple prediction models, including Random Forest (RF), Logistic Regression (LR), Support Vector Machine (SVM), extreme gradient boosting (XGB), and Recurrent Neural Network (RNN), to forecast the recurrence of candidemia. In Table 3, we present a comparative analysis of these models, demonstrating their performance metrics regarding accuracy, precision, recall, F1-score and Area Under the Curve (AUC). The ROC curves were constructed based on the AUC value (Fig 3). Our analysis demonstrated the effectiveness of all models, as each exhibited strong predictive performance with an AUC greater than 0.80. Notably, the RF model outperformed the others with an AUC of 0.87. The highest accuracy attained for predicting candidemia was 0.79 achieved by both the RF and RNN models.

Table 3. Performance of the machine-learning algorithms in internal validation cohort.

Model	Accuracy	Precision	Recall	F1 score	AUC
Logistic regression	0.73	0.69	0.78	0.74	0.82
Random forest	0.79	0.85	0.69	0.76	0.87
Support vector machine	0.73	0.69	0.78	0.74	0.82
Extreme Gradient boosting	0.76	0.79	0.69	0.73	0.83
Recurrent neural network	0.79	0.78	0.78	0.78	0.84

AUC: Area Under the Curve

Fig 3. Comparation of ROC curves among machine learning models for candidemia diagnostic in ICU patients. ROC: receiver operating characteristic curve; auc: area under the curve; LR: logistic regression; SVM: support vector machine; RF: random forest; XGB: extreme gradient boosting. RNN: recurrent neural network.

The calibration curves for our predictive models were presented in Fig 4. The Random Forest and Logistic Regression models showed excellent calibration, with their curves closely aligning with the diagonal line. The mean absolute errors (MAE) were 0.088 and 0.094, respectively. Likewise, the Recurrent Neural Network and Support Vector Machine models exhibit strong calibration performance. However, the calibration curve for the Extreme Gradient Boosting model noticeably deviates from diagonal line, suggesting a potential inaccuracy in comparison to the other models. In addition, we also provide the decision curves that assess the clinical usefulness of the RF model on the test set in Fig 5. These curves show that the RF model offers substantial benefit when the threshold probability of candidemia falls within the range of 55% to 85%.

Fig 4. Calibration curve for model validation of LR, RF, SVM, XGB and RNN on the testing set. The calibration curve plotted means of decile-binned predicted probabilities.

Fig 5. Decision curve analysis (DCA) of the random forest model on the test set. X-axis indicates the threshold probability for candidemia diagnosis and Y-axis indicates the net benefit. The red line represents the random forest model.

The Random Forest model continued to perform well again in our external validation cohort from Teng Zhou Central People's Hospital. As shown in Table 4, the RF model achieved a good predictive performance with an AUC of 0.83, slightly lower than the testing set. The RF model exhibited a precision of 0.83 and accuracy of 0.81 in the external validation cohort. These results confirm the model's reliability in correctly discriminating true positives and true negatives.

Table 4. Performance of Random Forest model in external validation cohort.

Model	Accuracy	Precision	Recall	F1	AUC
Logistic regression	0.73	0.63	0.63	0.63	0.80
Random forest	0.81	0.83	0.63	0.71	0.83
Support vector machine	0.71	0.59	0.63	0.61	0.78
Extreme Gradient boosting	0.80	0.68	0.81	0.74	0.83

Recurrent neural network	0.73	0.63	0.63	0.63	0.76

AUC: Area Under the Curve.

Interpretation of random forest predictive model

We used SHAP values to elucidate the contribution of individual features in the Random Forest model. As shown in Fig 6, all features are ranked by their importance. The most influential features in red are positioned at the top, while less significant ones in blue are at the bottom. There are a few key insights from SHAP analysis. First, total bilirubin emerges as the most crucial predictor for candidemia in the RF model, appearing that a higher level of total bilirubin is associated with an elevated risk of candidemia. Second, several other factors positively correlate with the risk of candidemia, including age, antimicrobial therapy, neutrophil count, use of immunosuppressive drugs, total parenteral nutrition, abdominal surgery, chemotherapy, the presence of a central venous catheter, a history of solid cancer, and duration of ICU stay. Third, lower levels of platelets, hemoglobin, lymphocytes, white blood cells, C-reactive protein, procalcitonin, and monocytes are negatively associated with the risk of candidemia.

Fig 6. Feature importance estimated using the Shapley additive explanations (SHAP) values. The plot sorts features by the sum of SHAP value magnitude over all samples. The blue to red color represents the feature value (red high, blue low). The x-axis measures the impacts on the model output (right positive, left negative).

The predictive nomogram for candidemia probability

In this study, we constructed a predictive nomogram based on 18 selected features to estimate the probability of candidemia. The primary goal of this analysis is to provide clinicians with a valuable resource for assessing and quantifying the likelihood of candidemia based on specifical clinical characteristics. As shown in Fig 7, each feature is assigned a probability score within a range from 0 to 100. The cumulative scores for all the features are then summed to generate a total score. This total score can then be projected onto the lower risk axis of the nomogram, enabling the prediction of the risk of candidemia diagnosis in ICU patients.

Fig 7. Nomogram of candidemia predicting using 18 clinical features. To use the nomogram, we first score each patient's variables to the score axis, added points of all the variables, draw a line from the total score axis to determine candidemia predicting probability on the lower line of the nomogram.

Discussion

In this study, we aimed to develop a predicative model for candidemia in intensive care unit patients using machine learning techniques. Our study cohort included 167 patients with candidemia infection and 167 patients with

bacteremia infection admitted to the ICU at Ji Ning NO.1 people's hospital from Jan. 2015 to Dec. 2022. Our approach distinguishes itself from prior researches that mainly focused on identifying risk factor for candidemia susceptibility, where healthy people or non-infected patients in medical ward served as the control group. Here, we not only identified risk factors but also constructed a diagnostic model, with bacteremia patients serving as the control model.

When compared our study with Li, et al.'s predictive model for candidemia in cancer patients [31], it is worth noting that while they achieved a slightly higher AUC value, variance in patient selection criteria plays a significant influence. Our study exclusively focused on ICU patients, a group with widely distributed risk factors. By using bacteremia patients within the same ICU as the control group in our model development, we aimed in order to better predicate the candidemia especially in ICU setting. Li et al. also employed bacteremia patients as the control group in their predictive model for invasive candidiasis [32], constituting 81.63% of candidemia and 64.79% of bacteremia cases from ICU, which yielded an AUC value of 0. 92. The slightly lower AUC values in this study may be attributed to the differences in the selected patient populations. An important distinction in our study is the use of data from an external hospital for validation, a practice not commonly observed in previous candidemia prediction study. This external validation provides additional credibility to the generalizability of our model.

We employed a range of machine learning algorithms, including LR, RF, SVM, XGB, and RNN, to construct diagnostic models using routine clinical data. These models displayed robust performance in distinguishing candidemia from bacteremia among ICU patients. Among these models, the RF model stood out as the most promising candidate. It achieved impressive precision, recall, and F1 scores of 0.85, 0.69, and 0.76, respectively. These metrics were either equal to or greater than the corresponding scores obtained by the RNN model (0.78). When subjected to external validation, the RF model maintained its excellence, yielding an accuracy of 0.81, precision of 0.83, recall of 0.63, and F1 score of 0.71 reflective of its exceptional diagnostic performance. The decision to select the Random Forest (RF) model as the final machine learning model was driven by its remarkable diagnostic performance, highlighted by a high AUC value of 0.87 and these outstanding evaluation metrics. In addition, RF algorithm displays an advantage in handling missing data points, whether they are numerical or factor variables. This capability is particularly valuable in the context of clinical studies, where missing data can be a common occurrence.

The Recurrent Neural Network (RNN) model demonstrated strong diagnostic performance as well, with AUC value of 0.84. This made it the second-highest performer, closely following the RF model. Notably, the RNN model shared the highest accuracy score of 0.79 with the RF model. As highlighted by Cleophas et al., RNN boasts several advantages, including the capacity to handle imperfect data and complex nonlinear relationships within the data set [33]. However, the RNN model did not demonstrate the best predictive performance, which may be due to the fact that the RNN algorithm is more suitable for large-scale data with time series. In contrast, the LR model exhibited comparatively lower diagnostic performance than RF and RNN, with an AUC value of 0.820. This observation aligns with the notion that the LR algorithm may not always be the ideal predictive model when handling with complex clinical data [34].

SHAP values were used to identify the crucial predictors of candidemia. Notably, the following factors emerged as positively associated with candidemia risk: high levels of total bilirubin, patient age, total parenteral nutrition (TPN), duration of ICU stay, the presence of a central venous catheter (CVC), and the influence of glucocorticoids. Among these factors, high level of total bilirubin is of most valuable feature. Serum bilirubin is a stable and well-established marker of liver impairment [35, 36]. The level of bilirubin is negatively correlated with antimicrobial immunity, rendering individuals more susceptible to candidiasis [37]. Albumin act as an antioxidant and free radical scavenger which can offer protection from inflammatory processes [38]. Patients with hypoalbuminemia are more susceptible to infect opportunistic pathogens like candidemia. However, hypoalbuminemia was not identified as a predict feature for distinguishing between candidemia and bacteremia in this model. In inflammatory states, capillary escape and breakdown of albumin increased which can lead to hypoalbuminemia [38]. Besides, server inflammation often combined with liver failure, which could decrease serum albumin mass because albumin synthesis rate is mainly decreased in liver failure [39]. Low serum albumin levels have been associated with an increased risk of mortality in patients with bloodstream infections because of bacterial or fungal infection [40]. Several studies have demonstrated a correlation between hypoalbuminemia and higher mortality rates in these patients. For example, a study by Lao *et al.* [41] found that low serum albumin levels were independently associated with increased mortality in patients with candidemia. Similarly, a study by A. Capelastegui *et al.* [42] showed that hypoalbuminemia was a significant predictor of mortality in patients with bacteremia.

The remaining positive predicators identified in our study have been supported by previous research in various contexts. For example, both TPN and CVC presence have been recognized as an independent risk factor for candidemia [2]. TPN induced candidemia may arise from alternations in gut microbiota [43], leading to changes in epithelial barrier function and subsequent intestinal inflammation [44]. The strong association of CVC presence with candidemia, even after adjusting for TPN, underscores its significance for candidemia [19].

Duration of stay in the ICU has also been proposed as an essential indicator of candidemia [45]. This metric is influenced by various factors, including patient demographics, treatment complexity and comorbidities. Moreover, glucocorticoids, which are used in the management of various immune diseases, carry several side effects, such as insulin resistance, increased susceptibility to severe infections, Cushing's syndrome, and peptic ulcers [46]. Prolonged treatment with high-dose systemic glucocorticoids elevates the risk of candidemia [47].

In addition, our study highlighted the association of recent abdominal surgery, chemotherapy, solid cancer and antimicrobial therapy with candidemia. Surgery may compromise the natural body barrier [48], cytotoxic chemotherapy can disrupt the mucosal integrity [49], and antimicrobial therapy may induce alterations in intestinal flora [2]. All these factors collectively create an environment conducive to Candida invasion through mesenteric circulation [2, 49].

Platelets are widely recognized as integral components of the host's defense system against invasive infections [50]. In our study, low platelet count was positively associated with candidemia prediction. This finding is significant since platelets paly a protective role in integrating vascular endothelial cells against Candida – induced damage [50]. The decreased presence of platelets may then increase the risk of candidemia. PCT and CRP are well-known for their

predictive value in assessing the prognosis of patients with candidiasis and bacterial infections. Some studies have even attempted to use these two markers to distinguish candidemia from bacteremia [51]. Like PCT and CRP, lymphocytes, WBC and monocytes were negatively correlated with candidemia in this study. These findings are particularly intriguing, as white blood play a pivotal role in initiation and maintenance of phagocyte-dependent fungal eliminations [52]. Furthermore, lymphocyte subtyping has even been used as a good marker in the early diagnosis of candidemia [53], which we did not included in this study. Interestingly, our study uncovers an association between low hemoglobin level and high level of bilirubin with an increased incidence of candidemia. This observation might be attributed to hemolytic factors produced by Candida, which can lead to rupture of red blood cells upon binding to the cell membrane[54].

This study has several clinical application prospects. Firstly, the developed machine learning models provide a reliable early prediction method for candidemia in intensive care unit (ICU) patients, which could assist clinicians in making timely decisions regarding the initiation of antifungal therapy. By identifying high-risk patients in advance, hospitals can arrange treatment and monitoring equipment in advance, thereby avoiding additional costs and adverse outcomes resulting from delayed diagnosis. Accurately identifying candidemia patients helps hospitals allocate resources more effectively and can reduce unnecessary resource wastage. Secondly, the identification of key risk factors through SHAP analysis reveals potential mechanisms underlying the development of candidemia, providing important insights for implementing personalized interventions for high-risk patients. Additionally, the predictive nomogram offers a convenient tool for clinicians to estimate the probability of candidemia based on patients' clinical characteristics, aiding in the optimization of individualized treatment plans. Overall, this study provides new insights and approaches for improving patient outcomes, optimizing resource utilization, and enhancing the management strategies for fungal bloodstream infections.

There are several limitations in our study. First, the sample size was constrained by the fact that the data was collected from a single medical center. The relatively small sample size may have implications for the generalizability of our findings. Second, our study design was retrospective in nature. This choice was necessitated by the low incidence of candidemia, which limited our ability to incorporate the latest biomarkers into our predictive model. Third, although our model demonstrated good performance and achieved acceptable results during external validation, it is essential to acknowledge that retrospective studies inherently carry the potential bias. Therefore, there is a need for multiple-center prospective cohorts to expand our understanding and refine the predicative model.

Conclusions

In this study, we have demonstrated that the machine learning model on the RF algorithm outperforms other algorithms in predicting candidemia. Our findings emphasize the critical importance of considering total bilirubin levels as a key predictor for candidemia, particularly among ICU patients. The RF model's predictive capabilities could serve as a valuable tool for clinicians in the early diagnosis and treatment of candidemia. The results of this study

suggest the potential utility of AI-assisted diagnosis for enhancing the clinical management of candidemia, representing a promising avenue for improving patient care in this context.

Author contributions

Funding acquisition：Qiang Meng, Feng Qu

Visualization：Qiang Meng

Writing – original draft：Qiang Meng, Zhi Jin

Conceptualization: Bowang Chen

Validation: Yingyuan Xu, Qiang Zhang

Data curation: Qiang Zhang, Zhi Jin

Software: Ranran Ding，Zhen Ma

Supervision: Shuhong Gao, Feng Qu

Funding

This research was funded by Key R&D Program of Jining, grant number 2023YXNS151.

Informed consent statement

The requirement for informed consent was waived because the retrospective nature of the study.

Data availability statement

Data available on request due to restrictions of ethical.

Conflicts of interest

The authors declare no conflict of interest.

References

1. Wisplinghoff H, Bischoff T, Tallent SM, Seifert H, Wenzel RP, Edmond MB. Nosocomial bloodstream infections in US hospitals: analysis of 24,179 cases from a prospective nationwide surveillance study. Clin Infect Dis. 2004;39(3):309-17. Epub 2004/08/13. doi: 10.1086/421946. PubMed PMID: 15306996.

2. Poissy J, Damonti L, Bignon A, Khanna N, Von Kietzell M, Boggian K, et al. Risk factors for candidemia: a prospective matched case-control study. Crit Care. 2020;24(1):109. Epub 2020/03/20. doi: 10.1186/s13054-020-2766-1. PubMed PMID: 32188500; PubMed Central PMCID: PMCPMC7081522.

3. Zaoutis TE, Argon J, Chu J, Berlin JA, Walsh TJ, Feudtner C. The epidemiology and attributable outcomes of candidemia in adults and children hospitalized in the United States: a propensity analysis. Clin Infect Dis. 2005;41(9):1232-9. Epub 2005/10/06. doi: 10.1086/496922. PubMed PMID: 16206095.

4. Fisher BT, Boge CLK, Xiao R, Shuster S, Chin-Quee D, Allen J, et al. Multicenter Prospective Study of Biomarkers for Diagnosis of Invasive Candidiasis in Children and Adolescents. Clin Infect Dis. 2022;75(2):248-59. Epub 2022/02/09. doi: 10.1093/cid/ciab928. PubMed PMID: 35134165; PubMed Central PMCID: PMCPMC9890499.

5. Pappas PG, Lionakis MS, Arendrup MC, Ostrosky-Zeichner L, Kullberg BJ. Invasive candidiasis. Nat Rev Dis Primers. 2018;4:18026. Epub 2018/05/12. doi: 10.1038/nrdp.2018.26. PubMed PMID: 29749387.

6. Bassetti M, Giacobbe DR, Vena A, Wolff M. Diagnosis and Treatment of Candidemia in the Intensive Care Unit. Semin Respir Crit Care Med. 2019;40(4):524-39. Epub 2019/10/05. doi: 10.1055/s-0039-1693704. PubMed PMID: 31585478.

7. Magadia RR, Weinstein MP. Laboratory diagnosis of bacteremia and fungemia. Infect Dis Clin North Am. 2001;15(4):1009-24. Epub 2002/01/10. doi: 10.1016/s0891-5520(05)70184-7. PubMed PMID: 11780265.

8. Playford EG, Lipman J, Jones M, Lau AF, Kabir M, Chen SC, et al. Problematic Dichotomization of Risk for Intensive Care Unit (ICU)-Acquired Invasive Candidiasis: Results Using a Risk-Predictive Model to Categorize 3 Levels of Risk From a Multicenter Prospective Cohort of Australian ICU Patients. Clin Infect Dis. 2016;63(11):1463-9. Epub 2016/09/08. doi: 10.1093/cid/ciw610. PubMed PMID: 27601224.

9. Morrell M, Fraser VJ, Kollef MH. Delaying the empiric treatment of candida bloodstream infection until positive blood culture results are obtained: a potential risk factor for hospital mortality. Antimicrobial agents and chemotherapy. 2005;49(9):3640-5. Epub 2005/08/30. doi: 10.1128/AAC.49.9.3640-3645.2005. PubMed PMID: 16127033; PubMed Central PMCID: PMCPMC1195428.

10. Zilberberg MD, Kollef MH, Arnold H, Labelle A, Micek ST, Kothari S, et al. Inappropriate empiric antifungal therapy for candidemia in the ICU and hospital resource utilization: a retrospective cohort study. BMC Infect Dis. 2010;10:150. Epub 2010/06/08. doi: 10.1186/1471-2334-10-150. PubMed PMID: 20525301; PubMed Central PMCID: PMCPMC2890008.

11. Liesenfeld O, Lehman L, Hunfeld KP, Kost G. Molecular diagnosis of sepsis: New aspects and recent developments. Eur J Microbiol Immunol (Bp). 2014;4(1):1-25. Epub 2014/03/29. doi: 10.1556/EuJMI.4.2014.1.1. PubMed PMID: 24678402; PubMed Central PMCID: PMCPMC3955828.

12. Zhang J, Cheng W, Li D, Chen J, Zhao G, Wang H, et al. Development and Validation of a Risk Score for Predicting Invasive Candidiasis in Intensive Care Unit Patients by Incorporating Clinical Risk Factors and Lymphocyte Subtyping. Front Cell Infect Microbiol. 2022;12:829066. Epub 2022/05/17. doi: 10.3389/fcimb.2022.829066. PubMed PMID: 35573797; PubMed Central PMCID: PMCPMC9091371.

13. Paiva JA, Charles PE. Biomarker-guided antifungal therapy in patients with suspected invasive candidiasis: Ready for prime time? Intensive Care Med. 2017;43(12):1889-91. Epub 2017/11/10. doi: 10.1007/s00134-017-4990-y. PubMed PMID: 29119220.

14. Posteraro B, Tumbarello M, De Pascale G, Liberto E, Vallecoccia MS, De Carolis E, et al. (1,3)-β-d-Glucan-based antifungal treatment in critically ill adults at high risk of candidaemia: an observational study. J Antimicrob Chemother. 2016;71(8):2262-9. Epub 2016/04/30. doi: 10.1093/jac/dkw112. PubMed PMID: 27125554.

15. Chertoff J, Ataya A. The Timing of Early Antibiotics and Hospital Mortality in Sepsis: Playing Devil's Advocate. Am J Respir Crit Care Med. 2017;196(7):934-5. Epub 2017/05/10. doi: 10.1164/rccm.201703-0657LE. PubMed PMID: 28485616.

16. Bhavani SV, Lonjers Z, Carey KA, Afshar M, Gilbert ER, Shah NS, et al. The Development and Validation of a Machine Learning Model to Predict Bacteremia and Fungemia in Hospitalized Patients Using Electronic Health Record Data. Crit Care Med. 2020;48(11):e1020-e8. Epub 2020/08/17. doi: 10.1097/CCM.0000000000004556. PubMed PMID: 32796184; PubMed Central PMCID: PMCPMC7554143.

17. Leiner T, Rueckert D, Suinesiaputra A, Baessler B, Nezafat R, Isgum I, et al. Machine learning in cardiovascular magnetic resonance: basic concepts and applications. J Cardiovasc Magn Reson. 2019;21(1):61. Epub 2019/10/09. doi: 10.1186/s12968-019-0575-y. PubMed PMID: 31590664; PubMed Central PMCID: PMCPMC6778980.

18. Yoo J, Kim SH, Hur S, Ha J, Huh K, Cha WC. Candidemia Risk Prediction (CanDETEC) Model for Patients With Malignancy: Model Development and Validation in a Single-Center Retrospective Study. JMIR Med Inform. 2021;9(7):e24651. Epub 2021/07/27. doi: 10.2196/24651. PubMed PMID: 34309570; PubMed Central PMCID: PMCPMC8367162.

19. Atamna A, Eliakim-Raz N, Mohana J, Ben-Zvi H, Sorek N, Shochat T, et al. Predicting candidemia in the internal medicine wards: a comparison with gram-negative bacteremia-a retrospectives study. Diagnostic microbiology and infectious disease. 2019;95(1):80-3. Epub 2019/05/28. doi: 10.1016/j.diagmicrobio.2019.04.007. PubMed PMID: 31129007.

20. Gao Y, Tang M, Li Y, Niu X, Li J, Fu C, et al. Machine-learning based prediction and analysis of prognostic risk factors in patients with candidemia and bacteraemia: a 5-year analysis. PeerJ. 2022;10. doi: 10.7717/peerj.13594.

21. Moons KG, Altman DG, Reitsma JB, Ioannidis JP, Macaskill P, Steyerberg EW, et al. Transparent Reporting of a multivariable prediction model for Individual Prognosis or Diagnosis (TRIPOD): explanation and elaboration. Ann Intern Med. 2015;162(1):W1-73. Epub 2015/01/07. doi: 10.7326/M14-0698. PubMed PMID: 25560730.

22. Ripoli A, Sozio E, Sbrana F, Bertolino G, Pallotto C, Cardinali G, et al. Personalized machine learning approach to predict candidemia in medical wards. Infection. 2020;48(5):749-59. Epub 2020/08/03. doi: 10.1007/s15010-020-01488-3. PubMed PMID: 32740866.

23. Remeseiro B, Bolon-Canedo V. A review of feature selection methods in medical applications. Comput Biol Med. 2019;112:103375. Epub 2019/08/06. doi: 10.1016/j.compbiomed.2019.103375. PubMed PMID: 31382212.

24. Lin H, Xue Y, Chen K, Zhong S, Chen L. Acute coronary syndrome risk prediction based on gradient boosted tree feature selection and recursive feature elimination: A dataset-specific modeling study. PLoS One. 2022;17(11):e0278217. Epub 2022/11/30. doi: 10.1371/journal.pone.0278217. PubMed PMID: 36445881; PubMed Central PMCID: PMCPMC9707772.

25. Guyon I, Elisseeff A. An Introduction to Variable and Feature Selection. Journal of machine learning research. 2003;(3):1157-82.

26. Zeng Z, Tang X, Liu Y, He Z, Gong X. Interpretable recurrent neural network models for dynamic prediction of the extubation failure risk in patients with invasive mechanical ventilation in the intensive care unit. BioData Min. 2022;15(1):21. Epub 2022/09/27. doi: 10.1186/s13040-022-00309-7. PubMed PMID: 36163063; PubMed Central PMCID: PMCPMC9513908.

27. Alba AC, Agoritsas T, Walsh M, Hanna S, Iorio A, Devereaux PJ, et al. Discrimination and Calibration of Clinical Prediction Models: Users' Guides to the Medical Literature. JAMA. 2017;318(14):1377-84. Epub 2017/10/20. doi: 10.1001/jama.2017.12126. PubMed PMID: 29049590.

28. Li J, Gong M, Joshi Y, Sun L, Huang L, Fan R, et al. Machine Learning Prediction Model for Acute Renal Failure After Acute Aortic Syndrome Surgery. Frontiers in medicine. 2021;8:728521. Epub 2022/02/04. doi: 10.3389/fmed.2021.728521. PubMed PMID: 35111767; PubMed Central PMCID: PMCPMC8801502.

29. Giorgi FM, Ceraolo C, Mercatelli D. The R Language: An Engine for Bioinformatics and Data Science. Life (Basel). 2022;12(5). Epub 2022/05/29. doi: 10.3390/life12050648. PubMed PMID: 35629316; PubMed Central PMCID: PMCPMC9148156.

30. Deberneh HM, Kim I. Prediction of Type 2 Diabetes Based on Machine Learning Algorithm. Int J Environ Res Public Health. 2021;18(6). Epub 2021/04/04. doi: 10.3390/ijerph18063317. PubMed PMID: 33806973; PubMed Central PMCID: PMCPMC8004981.

31. Li J, Li Y, Gao Y, Niu X, Tang M, Fu C, et al. Prediction of Prognostic Risk Factors in Patients with Invasive Candidiasis and Cancer: A Single-Centre Retrospective Study. BioMed Research International. 2022;2022:1-9. doi: 10.1155/2022/7896218.

32. Li Y, Wu Y, Gao Y, Niu X, Li J, Tang M, et al. Machine-learning based prediction of prognostic risk factors in patients with invasive candidiasis infection and bacterial bloodstream infection: a singled centered retrospective study. BMC Infect Dis. 2022;22(1):150. Epub 2022/02/15. doi: 10.1186/s12879-022-07125-8. PubMed PMID: 35152879; PubMed Central PMCID: PMCPMC8841094.

33. Cleophas TJ, Cleophas TF. Artificial intelligence for diagnostic purposes: principles, procedures and limitations. cclm. 2010;48(2):159-65. doi: 10.1515/cclm.2010.045.

34. Holmes JH, Sacchi L, Bellazzi R, Peek N. Artificial Intelligence in Medicine AIME 2015. Artif Intell Med. 2017;81:1-2. Epub 2017/07/25. doi: 10.1016/j.artmed.2017.06.011. PubMed PMID: 28733119.

35. Perez Ruiz de Garibay A, Kortgen A, Leonhardt J, Zipprich A, Bauer M. Critical care hepatology: definitions, incidence, prognosis and role of liver failure in critically ill patients. Critical Care. 2022;26(1). doi: 10.1186/s13054-022-04163-1.

36. Hadem J, Stiefel P, Bahr MJ, Tillmann HL, Rifai K, Klempnauer J, et al. Prognostic implications of lactate, bilirubin, and etiology in German patients with acute liver failure. Clin Gastroenterol Hepatol. 2008;6(3):339-45. Epub 2008/03/11. doi: 10.1016/j.cgh.2007.12.039. PubMed PMID: 18328438.

37. Chang Y-C, Chen J-S, Yin C-H, Shin-Jung Lee S, Chen W-C. Candidemia in hospitalized cirrhotic patients with bloodstream infection: A retrospective analysis and brief summary of published studies. Journal of the Chinese Medical Association. 2022;85(3):295-303. doi: 10.1097/jcma.0000000000000695.

38. Soeters PB, Wolfe RR, Shenkin A. Hypoalbuminemia: Pathogenesis and Clinical Significance. JPEN J Parenter Enteral Nutr. 2019;43(2):181-93. Epub 2018/10/06. doi: 10.1002/jpen.1451. PubMed PMID: 30288759; PubMed Central PMCID: PMCPMC7379941.

39. Rafferty MJ, Mcmillan DC, Preston TC, Hamid R, Small AC, Joshi N, et al. Reprioritisation of Liver Export Protein Synthesis in Patients with Decompensated Alcoholic Liver Disease. 2016;(3).

40. Wiedermann CJ. Hypoalbuminemia as Surrogate and Culprit of Infections. Int J Mol Sci. 2021;22(9). Epub 2021/05/01. doi: 10.3390/ijms22094496. PubMed PMID: 33925831; PubMed Central PMCID: PMCPMC8123513.

41. Lao M, Li C, Li J, Chen D, Ding M, Gong Y. Opportunistic invasive fungal disease in patients with type 2 diabetes mellitus from Southern China: Clinical features and associated factors. Journal of diabetes investigation. 2020;11(3):731-44. Epub 2019/11/24. doi: 10.1111/jdi.13183. PubMed PMID: 31758642; PubMed Central PMCID: PMCPMC7232281.

42. Capelastegui A, Zalacain R, Bilbao A, Egurrola M, Iturriaga LA, Quintana JM, et al. Pneumococcal pneumonia: differences according to blood culture results. BMC Pulm Med. 2014;14:128. Epub 2014/08/07. doi: 10.1186/1471-2466-14-128. PubMed PMID: 25096919; PubMed Central PMCID: PMCPMC4127038.

43. Demehri FR, Barrett M, Ralls MW, Miyasaka EA, Feng Y, Teitelbaum DH. Intestinal epithelial cell apoptosis and loss of barrier function in the setting of altered microbiota with enteral nutrient deprivation. Front Cell Infect Microbiol. 2013;3:105. Epub 2014/01/07. doi: 10.3389/fcimb.2013.00105. PubMed PMID: 24392360; PubMed Central PMCID: PMCPMC3870295.

44. Harris JK, El Kasmi KC, Anderson AL, Devereaux MW, Fillon SA, Robertson CE, et al. Specific microbiome changes in a mouse model of parenteral nutrition associated liver injury and intestinal inflammation. PLoS One. 2014;9(10):e110396. Epub 2014/10/21. doi: 10.1371/journal.pone.0110396. PubMed PMID: 25329595; PubMed Central PMCID: PMCPMC4203793.

45. Zhang Z, Zhu R, Luan Z, Ma X. Risk of invasive candidiasis with prolonged duration of ICU stay: a systematic review and meta-analysis. BMJ Open. 2020;10(7):e036452. Epub 2020/07/15. doi: 10.1136/bmjopen-2019-036452. PubMed PMID: 32660950; PubMed Central PMCID: PMCPMC7359383.

46. Yang R, Yu Y. Glucocorticoids are double-edged sword in the treatment of COVID-19 and cancers. Int J Biol Sci. 2021;17(6):1530-7. Epub 2021/04/29. doi: 10.7150/ijbs.58695. PubMed PMID: 33907516; PubMed Central PMCID: PMCPMC8071771.

47. Lionakis MS, Kontoyiannis DP. Glucocorticoids and invasive fungal infections. Lancet. 2003;362(9398):1828-38. Epub 2003/12/05. doi: 10.1016/s0140-6736(03)14904-5. PubMed PMID: 14654323.

48. Teoh F, Pavelka N. How Chemotherapy Increases the Risk of Systemic Candidiasis in Cancer Patients: Current Paradigm and Future Directions. Pathogens. 2016;5(1). Epub 2016/01/20. doi: 10.3390/pathogens5010006. PubMed PMID: 26784236; PubMed Central PMCID: PMCPMC4810127.
49. Nucci M, Anaissie E. Should vascular catheters be removed from all patients with candidemia? An evidence-based review. Clin Infect Dis. 2002;34(5):591-9. Epub 2002/01/26. doi: 10.1086/338714. PubMed PMID: 11810600.
50. Yeaman MR, Cheng D, Desai B, Kupferwasser LI, Xiong YQ, Gank KD, et al. Susceptibility to thrombin-induced platelet microbicidal protein is associated with increased fluconazole efficacy against experimental endocarditis due to Candida albicans. Antimicrobial agents and chemotherapy. 2004;48(8):3051-6. Epub 2004/07/27. doi: 10.1128/AAC.48.8.3051-3056.2004. PubMed PMID: 15273120; PubMed Central PMCID: PMCPMC478484.
51. Wang Q, Wang C, Yang M, Li X, Cui J, Wang C. Diagnostic efficacy of serum cytokines and chemokines in patients with candidemia and bacteremia. Cytokine. 2020;130:155081. Epub 2020/04/05. doi: 10.1016/j.cyto.2020.155081. PubMed PMID: 32247169.
52. Hatinguais R, Willment JA, Brown GD. PAMPs of the Fungal Cell Wall and Mammalian PRRs. Curr Top Microbiol Immunol. 2020;425:187-223. Epub 2020/03/18. doi: 10.1007/82_2020_201. PubMed PMID: 32180018.
53. Zhang J, Cui N, Wang H, Han W, Li Y, Xiao M, et al. Invasive Fungal Disease in Critically Ill Patients at High Risk: Usefulness of Lymphocyte Subtyping. J Intensive Care Med. 2020;35(9):909-18. Epub 2018/09/21. doi: 10.1177/0885066618800690. PubMed PMID: 30231674.
54. Roy U, Kornitzer D. Heme-iron acquisition in fungi. Current opinion in microbiology. 2019;52:77-83. Epub 2019/07/03. doi: 10.1016/j.mib.2019.05.006. PubMed PMID: 31265986.

Supporting information

S1 Table. Characteristics of selected features in the external validation group. Definition of abbreviations: y: years; TPN: Total parenteral nutrition; CVC: central venous catheter; WBC: white blood cell; PCT: procalcitonin; CRP: C-reactive protein; ICU: intensive care unit; IQR: interquartile range; SD: standard deviation; *The t-test for metric variable if data are normally distributed; **the Chi-square test (big sample size) and Fisher's exact test (small sample size) for categorical variables; ***the Mann–Whitney U-test for metric variables if data are not normally distributed.

S2 Table. Comparison of selected features between internal and external validation. Definition of abbreviations: y: years; TPN: Total parenteral nutrition; CVC: central venous catheter; WBC: white blood cell; PCT: procalcitonin; CRP: C-reactive protein; ICU: intensive care unit; IQR: interquartile range; SD: standard deviation; *The t-test for metric variable if data are normally distributed; **the Chi-square test (big sample size) and Fisher's exact test (small sample size) for categorical variables; ***the Mann–Whitney U-test for metric variables if data are not normally distributed.

Figure

Click here to access/download;Figure;Fig1.tif.tif

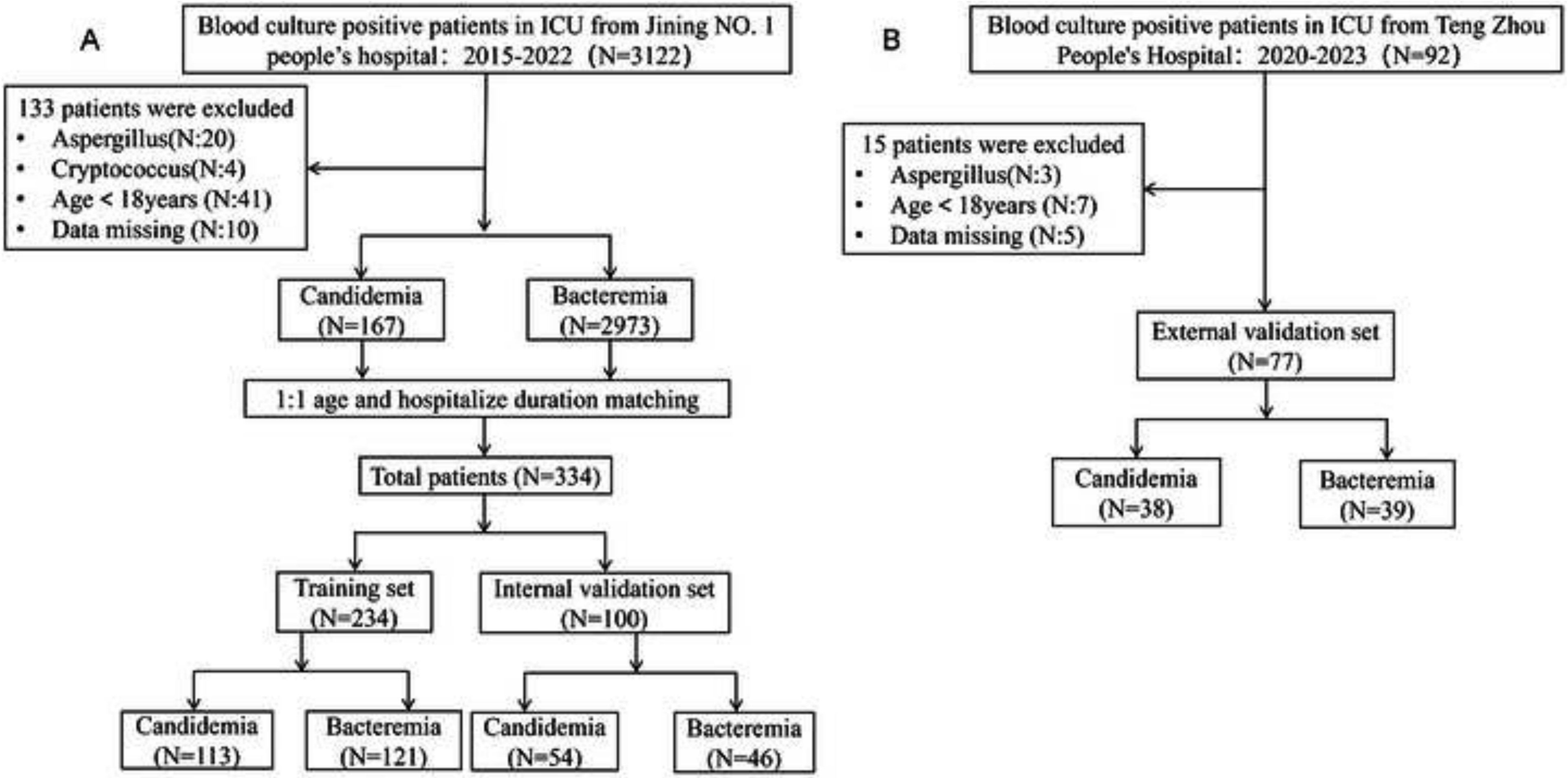

Click here to access/download;Figure;Fig2.tif.tif

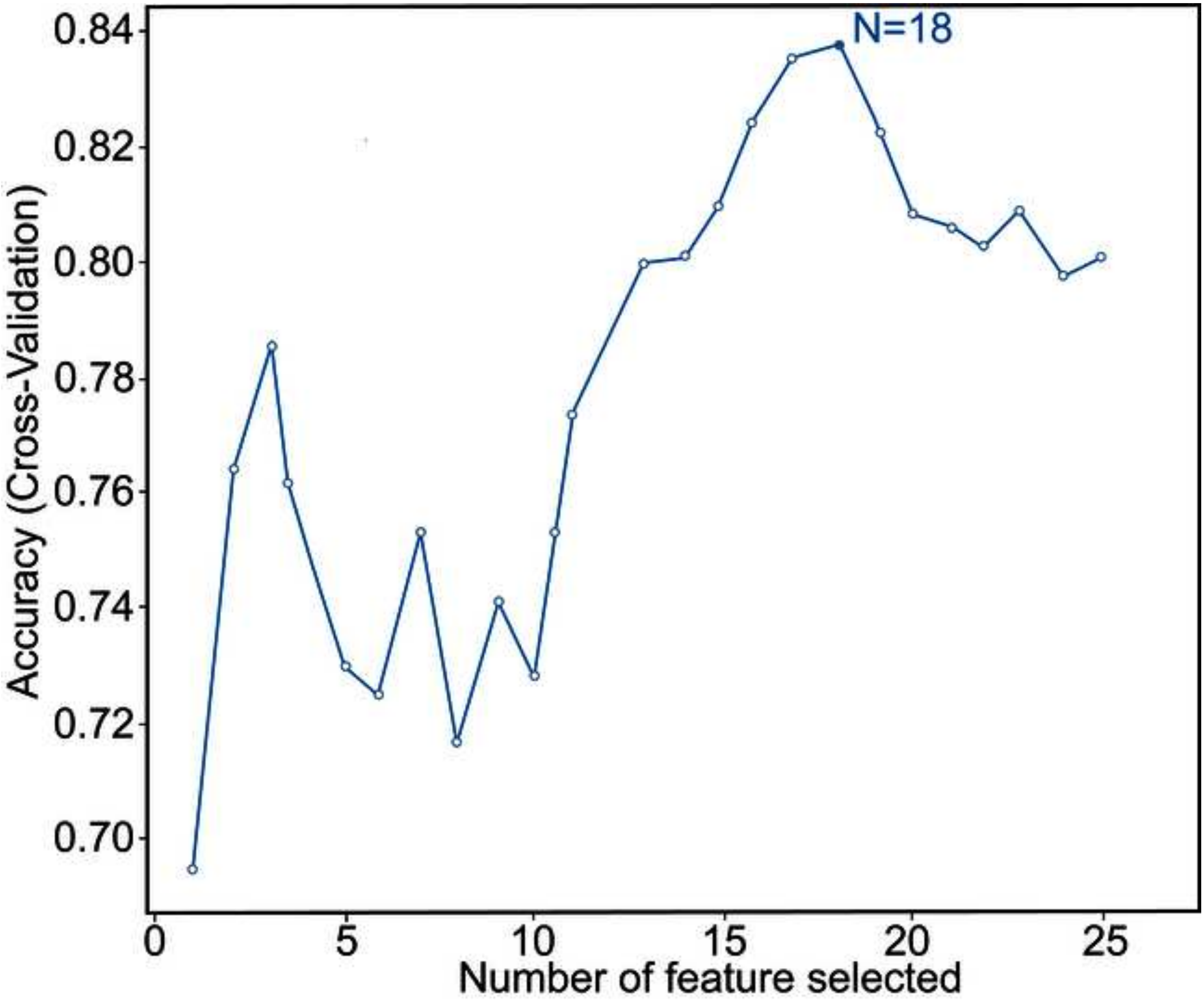

Figure

Click here to access/download;Figure;Fig3.tif.tif

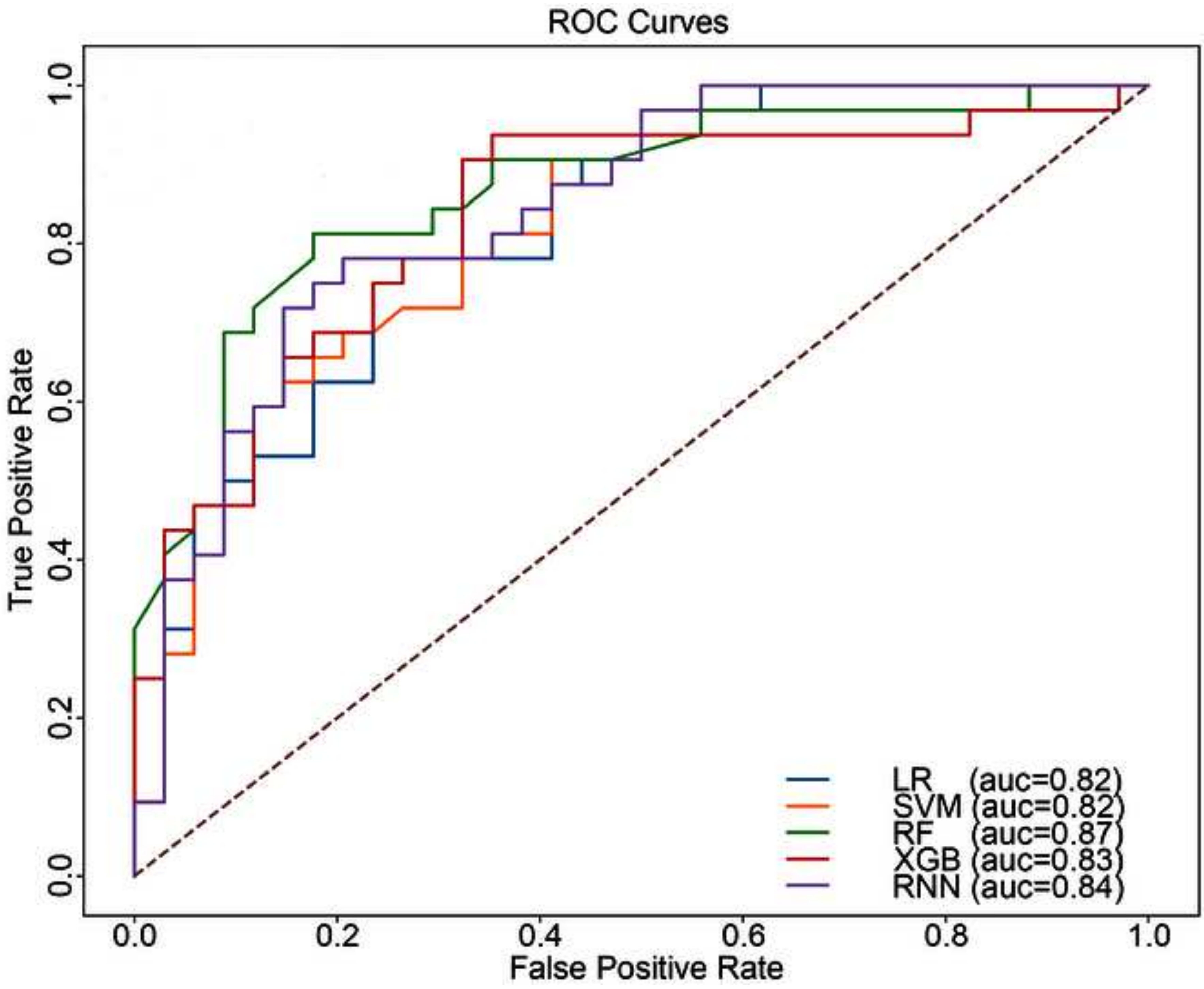

Figure
Click here to access/download;Figure;Fig 4.tif.tif

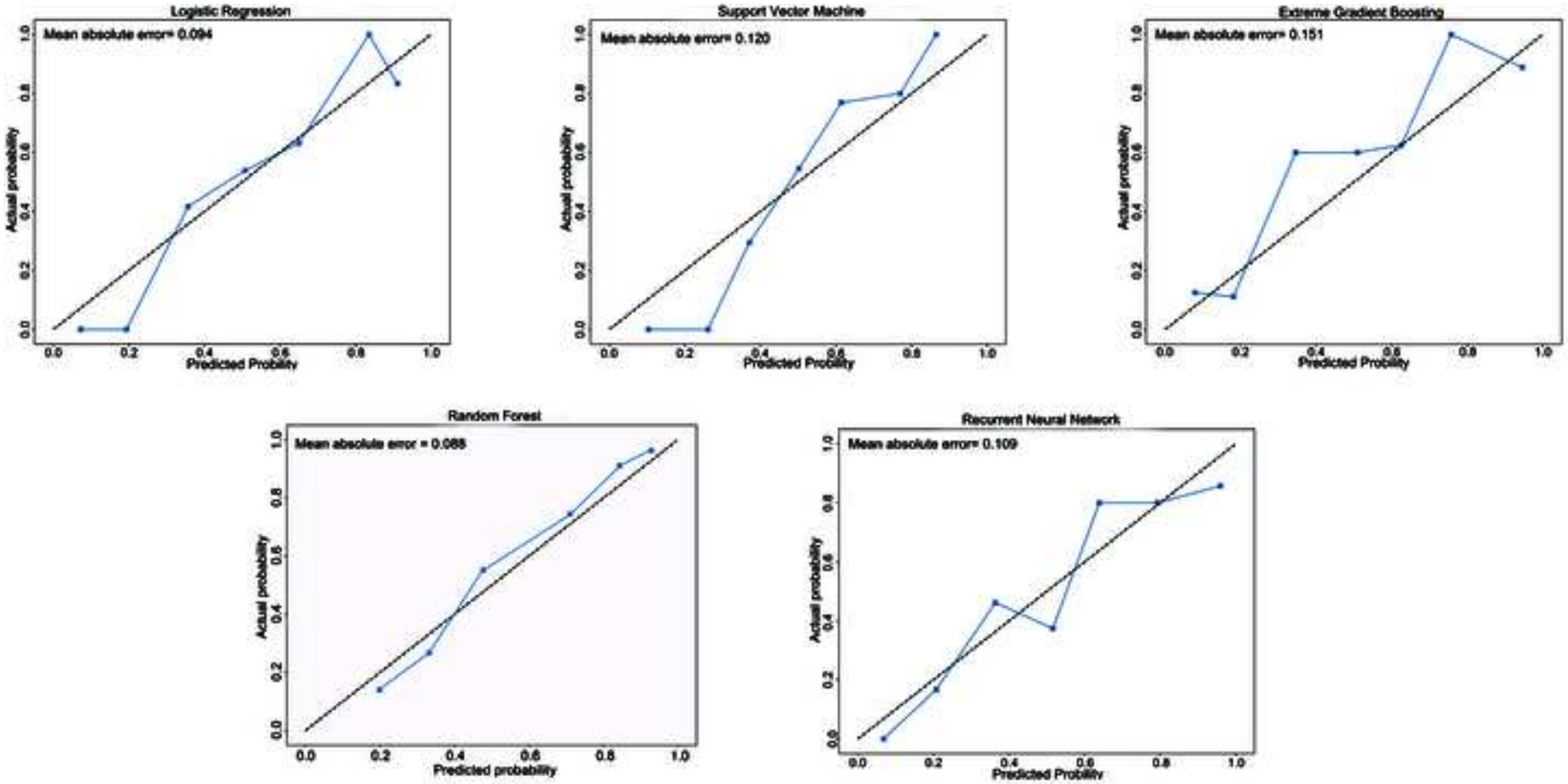

 Click here to access/download;Figure;Fig5.tif.tif

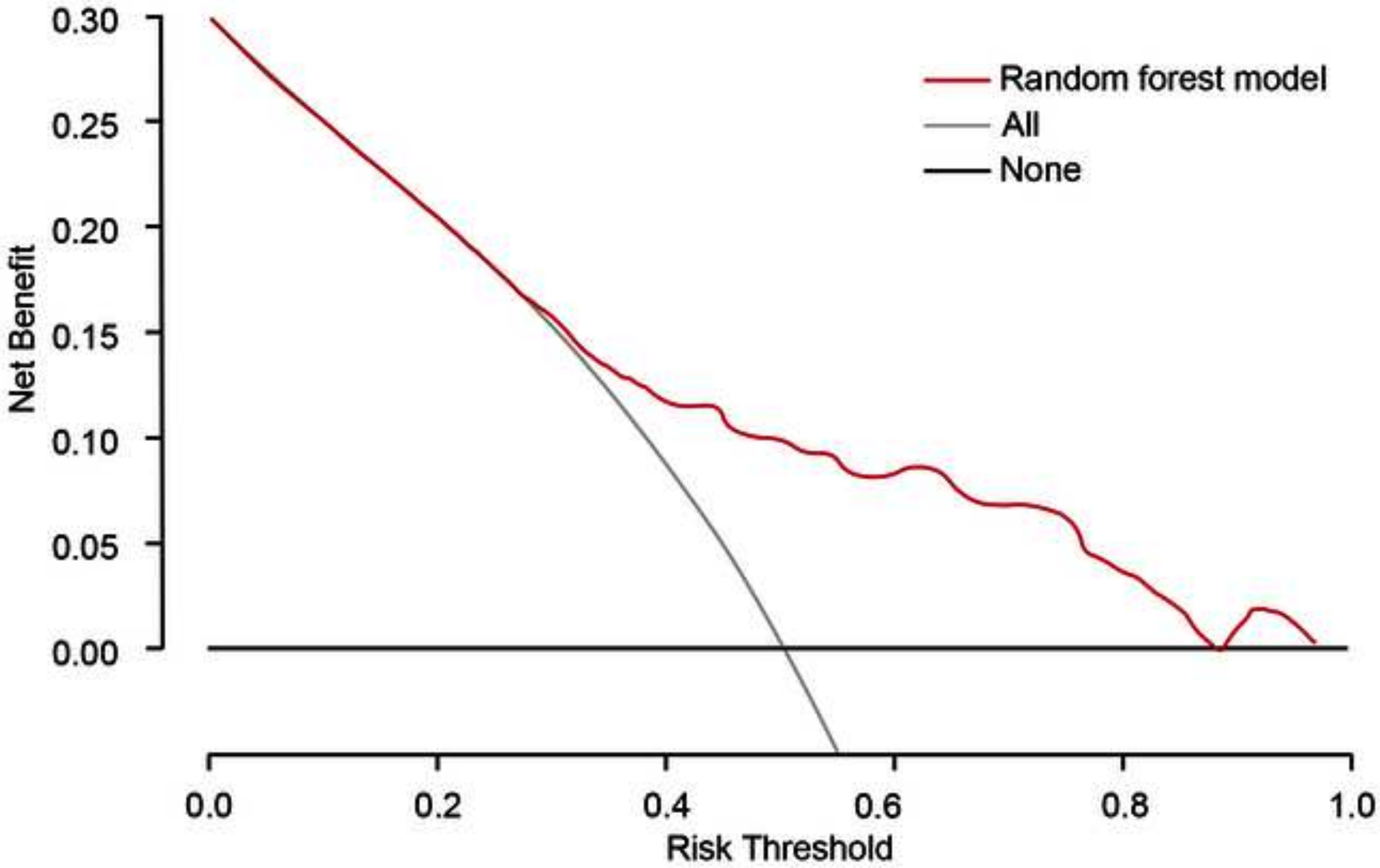

 Click here to access/download;Figure;Fig6.tif.tif

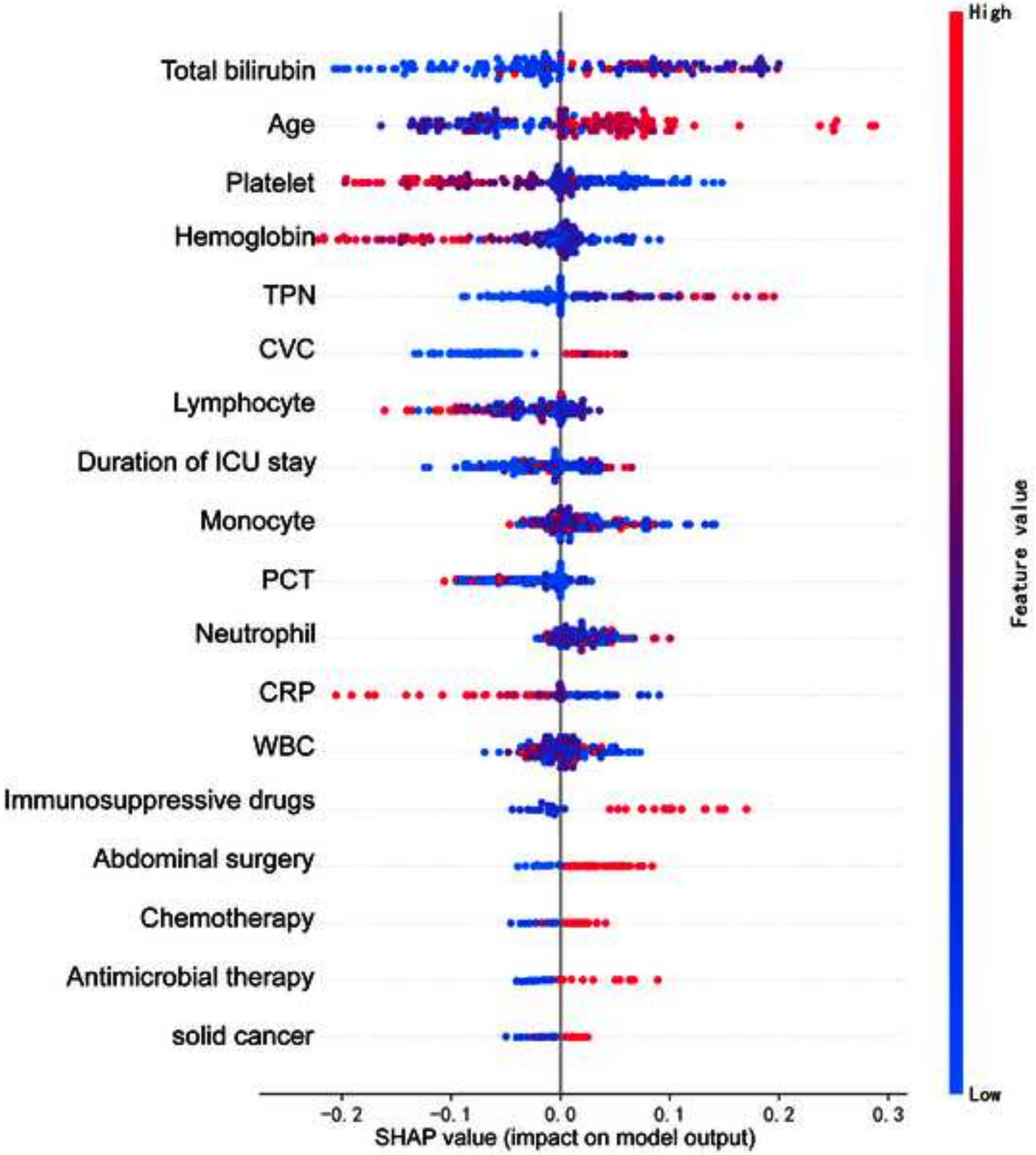

Figure

Click here to access/download;Figure;Fig7.tif.tif

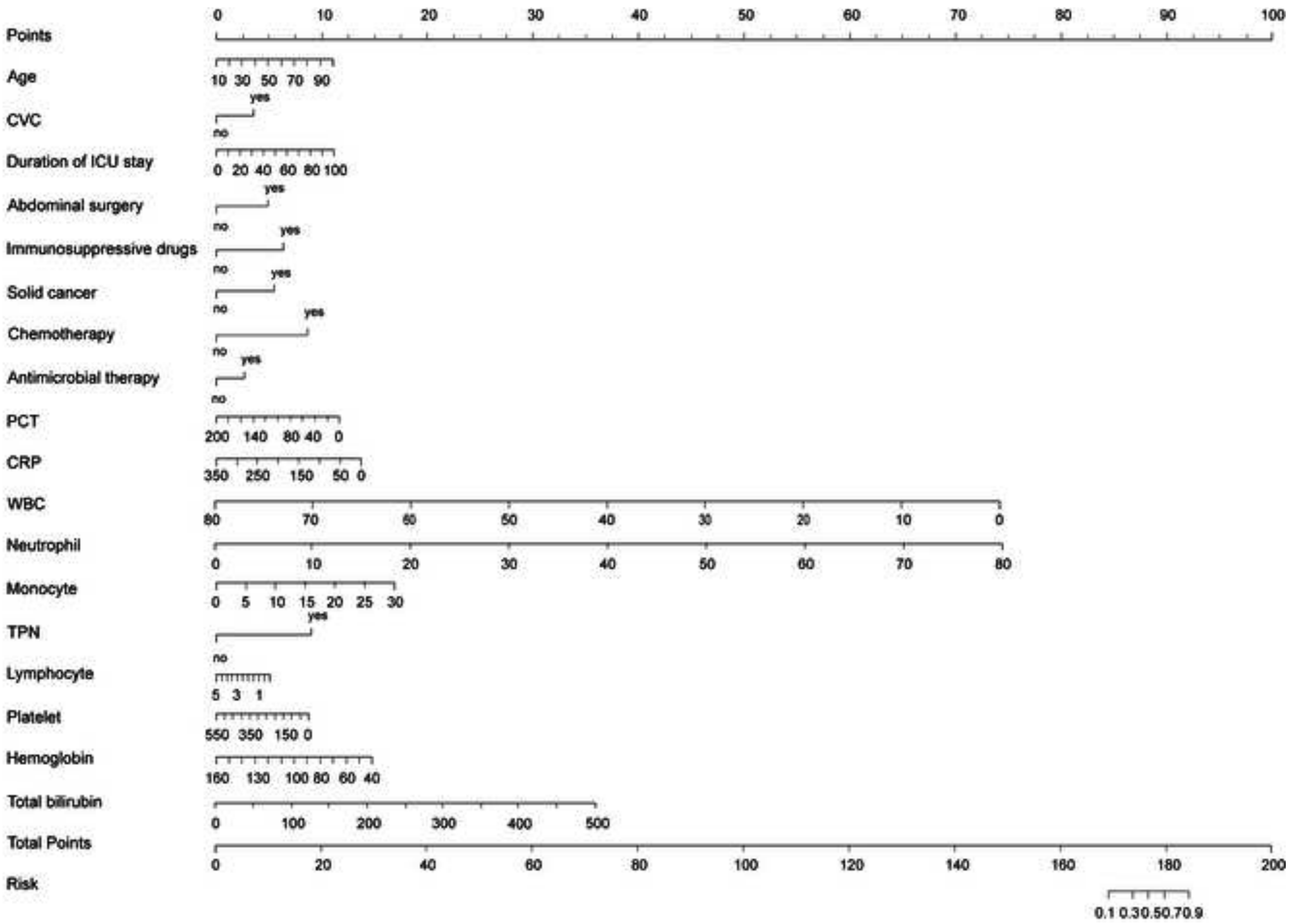

Click here to access/download
Supporting Information
S1 Table.docx

Click here to access/download
Supporting Information
S2 Table.docx
